# Carotid Phase-Contrast Magnetic Resonance before Treatment: 4D-Flow versus Standard 2D Imaging

**DOI:** 10.3390/tomography7040044

**Published:** 2021-09-28

**Authors:** Francesco Secchi, Caterina Beatrice Monti, Davide Capra, Renato Vitale, Daniela Mazzaccaro, Michele Conti, Ning Jin, Daniel Giese, Giovanni Nano, Francesco Sardanelli, Massimiliano M. Marrocco-Trischitta

**Affiliations:** 1 Department of Biomedical Sciences for Health, Università degli Studi di Milano, 20100 Milan, Italy; caterina.monti@unimi.it (C.B.M.); davide.capra@unimi.it (D.C.); renato.vitale@unimi.it (R.V.); giovanni.nano@unimi.it (G.N.); f.sardanelli@grupposandonato.it (F.S.); 2 Unit of Radiology, IRCCS Policlinico San Donato, 20097 San Donato Milanese, Italy; 3 Unit of Vascular Surgery, IRCCS Policlinico San Donato, 20097 San Donato Milanese, Italy; daniela.mazzaccaro@grupposandonato.it (D.M.); Massimiliano.MarroccoTrischitta@grupposandonato.it (M.M.M.-T.); 4 Department of Civil Engineering and Architecture, University of Pavia, 27100 Pavia, Italy; michele.conti@unipv.it; 5 Siemens Medical Solutions USA, Inc., Malvern, PA 19355, USA; ning.jin@siemens-healthineers.com; 6 Magnetic Resonance, Siemens Healthcare GmbH, 91052 Erlangen, Germany; giese.daniel@siemens-healthineers.com; 7 Clinical Research Unit, Cardiovascular Department, IRCCS Policlinico San Donato, 20097 San Donato Milanese, Italy

**Keywords:** magnetic resonance imaging, carotid stenosis, endarterectomy, carotid arteries, randomized controlled trial, phase-contrast magnetic resonance

## Abstract

The purpose of this study was to evaluate the level of agreement between flow/velocity data obtained from 2D-phase-contrast (PC) and 4D-flow in patients scheduled for treatment of carotid artery stenosis. Image acquisition was performed using a 1.5 T scanner. We compared mean flow rates, vessel areas, and peak velocities obtained during the acquisition with both techniques in 20 consecutive patients, 15 males and 5 females aged 69 ± 5 years (mean ± standard deviation). There was a good correlation between both techniques for the CCA flow (*r* = 0.65, *p* < 0.001), whereas for the ICA flow and ECA flow the correlation was only moderate (*r* = 0.4, *p* = 0.011 and *r* = 0.45, *p* = 0.003, respectively). Correlations of peak velocities between methods were good for CCA (*r* = 0.56, *p* < 0.001) and moderate for ECA (*r* = 0.41, *p* = 0.008). There was no correlation for ICA (*r* = 0.04, *p* = 0.805). Cross-sectional area values between methods showed no significant correlations for CCA (*r* = 0.18, *p* = 0.269), ICA (*r* = 0.1, *p* = 0.543), and ECA (*r* = 0.05, *p* = 0.767). Conclusion: the 4D-flow imaging provided a good correlation of CCA and a moderate correlation of ICA flow rates against 2D-PC, underestimating peak velocities and overestimating cross-sectional areas in all carotid segments.

## 1. Introduction

Carotid artery stenosis due to atherosclerotic plaques is one of the major causes of ischemic stroke [1]. Carotid endarterectomy (CEA) was established as safe and effective for reducing this risk by randomized controlled trials that were conducted approximately 30 years ago. Current guidelines for the treatment of carotid stenosis, which are still based on the evidence reported from those trials, recommend surgical revascularization in patients with a high degree of carotid stenosis [2]. In current clinical practice, duplex ultrasound (DUS) and the computed tomography angiography (CTA) represent the main imaging modalities for evaluating luminal carotid stenosis [3].

Carotid bi-dimensional DUS can reliably detect severe internal carotid artery stenosis using established criteria, which are based on the manual sampling of turbulent flow within the stenosis [4]. According to these criteria, the identification of a peak systolic velocity > 230 cm/s can reliably predict the presence of a stenosis > 70%. Nevertheless, DUS is highly dependent on readers’ expertise, which can affect the reproducibility of such a technique [5]. Furthermore, the anatomical features of the carotid plaque, such as distal/proximal localization or the presence of calcifications inducing acoustic shadows, may limit the accuracy of the assessment [6]. Finally, DUS provides bi-dimensional images that may offer an incomplete evaluation of the luminal stenosis.

CTA may help overcome the limits of DUS and, therefore, represents the reference standard for the measurement of the degree of stenosis [2]. Nevertheless, CTA requires the administration of iodinate contrast medium, exposes the patients to ionizing radiations, and does not provide information about the hemodynamic features of the stenosis.

Magnetic resonance (MR) recently emerged as a leading non-invasive, in vivo imaging modality for both plaque morphology [7] and hemodynamic evaluation of the stenosis. Currently, MR flow assessment is based predominantly on two-dimensional phase-contrast (2D-PC) sequences [8,9], which enable the calculation of velocities, flow rates, and blood volumes passing through a single fixed plane. As a major limitation, the desired analysis plane must be established a priori, and no additional analyses for other planes are possible after the 2D-PC acquisition [10]. The use of a three-dimensional (3D) time-resolved sequence (4D-flow), could provide a more precise assessment of 3D blood flow in vivo [11,12], hypothetically overcoming the limitations of DUS and 2D-PC, and sparing the patients from ionizing radiation exposure and contrast medium administration. However, data about the accuracy of 4D-flow MR are still lacking.

Hence, the purpose of our study was to evaluate the level of agreement between flow/velocity data obtained from 2D-PC and from 4D-flow in pre-procedure side and non-procedure side in patients scheduled for treatment of carotid artery stenosis.

## 2. Materials and Methods

### 2.1. Patient Selection

Twenty consecutive patients with unilateral carotid stenosis enrolled in the BAROX trial (ClinicalTrials.gov registration number: NCT03493971) were included in this study. The “BAROX” trial is a monocentric randomized controlled trial, which enrolls patients younger than 75 years, affected by symptomatic internal carotid artery (ICA) stenosis ≥ 70% or asymptomatic ICA stenosis ≥ 80% (based on the European Carotid Surgery Trial (ECST) criteria), who are randomized to receive either CEA or carotid stenting. The “BAROX” trial received funding from the Italian Ministry of Health for Research (PE-2013-02355484) and approval of the local ethical committee (record number 62/int/2017, 8 June 2017). All patients gave written informed consent.

According to the protocol, patients with contralateral carotid occlusion or ≥70% stenosis were excluded. Likewise, patients with bilateral carotid stenosis ≥ 70% were excluded. As a part of the study protocol, patients underwent 2D-PC and 4D-flow of the neck arteries in order to assess flow characteristics of carotid bifurcations. For each patient, MR images were acquired on both carotid arteries.

### 2.2. MR Acquisition

Image acquisition was performed using a 1.5 T scanner (MAGNETOM Aera, Siemens Healthcare, Erlangen, Germany) using a 20-channel head-neck coil. The duration of the entire imaging protocol, including a localizer sequence, the 2D-PC, and 4D-flow acquisitions, was approximately 20 min.

Two 2D-PC scanning planes were positioned perpendicular to the axis of the vessel. The first slice was placed below the carotid bifurcation in a plane so that both common carotid arteries were visible, and the second was placed right above the bifurcation where the internal and external carotid arteries were orthogonal to the acquisition plane, ensuring that no collateral vessels emerged proximally. The following technical parameters were chosen: time of echo (TE) 2.8–3.2 ms, echo spacing 5.3 ms, segment number 4, temporal resolution 40.2 ms, flip angle 20°, bandwidth 445 Hz/pixel, field of view (FOV) 103 × 150 mm^2^, acquired in-plane resolution 1.25 × 1.13 mm^2^, slice thickness 6 mm, and velocity encoding (VENC) 80 cm/s. In presence of aliasing artefact, VENC was increased up to 150 cm/s.

The 4D-flow technique consisted of a flow-sensitive 3D time-resolved phase-contrast sequence with retrospective electrocardiogram gating yielding the time-dependent 3D velocity field in the head and neck arteries (WIP 785B). Acquisition parameters were: TE 2.3–3.1 ms, echo spacing 5.1 ms, flip angle 8°, segment number 2, temporal resolution 40.6–43.4 ms, bandwidth 490 Hz/pixel, FOV 340–232 mm^2^, 3D acquired resolution 3.5 × 2.4 × 3.9 mm, and VENC 80 cm/s. Images were acquired in a coronal plane, yielding 28 to 52 slices depending on the patient size.

### 2.3. Post-Processing

In order to assess the differences between imaging techniques, we compared mean flow rates, mean vessel areas, and peak velocities obtained during the acquisition.

**Figure 1:**
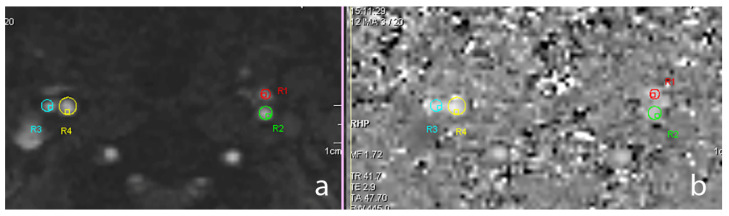
The 2D magnitude (**a**) and phase contrast (**b**) slice above the carotid bifurcation showing the cross-sectional areas in the regions of interest. R1: left external carotid artery. R2: left internal carotid artery. R3: right external carotid artery. R4: right internal carotid artery.

The 2D-PC data were processed with the Argus software version VE30A (Siemens Healthineers, Erlangen, Germany) allowing the user to select the vessel of interest in the magnitude images and then retrieve, from PC images, the instantaneous flow rate and vessel cross-sectional areas at each timestep, as well as the peak velocity found during the entire cardiac cycle. The software automatically outputs the mean flow rate and average cross-sectional area by averaging the two variables across time. Figure 1 shows a magnitude 2D-PC slice placed above the carotid bifurcation with the values of the cross-sectional areas in the external carotid artery (ECA) and ICA.

**Figure 2:**
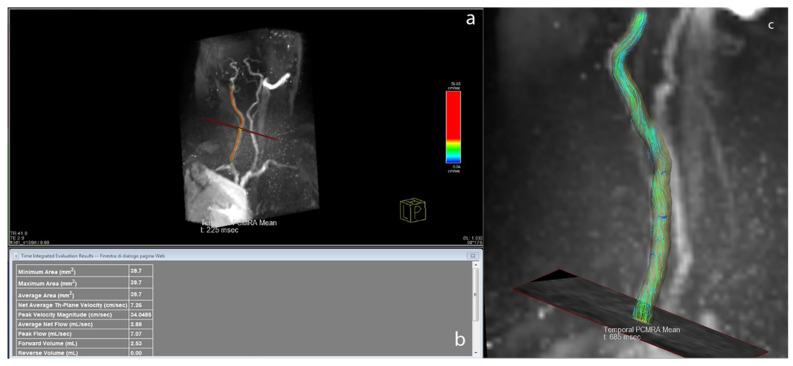
Reconstruction of a carotid artery using 4D-flow software; (**a**) plane of interest is highlighted; (**b**) table with flow data from the plane shown in a; and (**c**) single frame from streamlines of common carotid artery and internal carotid artery.

The 4D-flow data were processed with the prototype software 4D Flow Demonstrator v2.4 (Siemens Healthineers, Erlangen, Germany). In a first step, the entire slab was loaded, and background phase correction and antialiasing were applied. Then the user selected the vessel of interest on the magnitude images, where a centreline was automatically calculated. The region of interest included the visible portions of the common carotid artery (CCA) and the origin of the ICA and ECA after the bifurcation. Then, the user placed a plane orthogonal to the vessel centreline to retrieve flow characteristics. Figure 2 illustrates the flow plane positioning and the flow streamlines in the 4D-flow acquisitions. Three planes were selected with the aim of reproducing the same positions of the 2D-PC slices acquired in the CCA, ICA, and ECA.

### 2.4. Statistical Analysis

Each variable of interest was tested for normality using the Shapiro-Wilk test. Differences were compared using the paired *t*-test or the Wilcoxon matched-pairs signed-rank test for normally and non-normally distributed samples, respectively. Statistical significance was considered for *p* < 0.05.

Mean flow rate, peak velocity, and average cross-sectional areas were normally distributed and compared by calculating the Pearson *r* correlation coefficient between both acquisition methods. Statistically significant correlations were interpreted according to Cohen [13].

Bland-Altman plots were produced in order to visualize the level of agreement between 4D-flow and 2D-PC. The differences between imaging modalities were plotted against their mean values. All statistical analyses were performed with GraphPad Prism v8 (GraphPad Software, La Jolla, CA, USA).

## 3. Results

The 20 consecutive patients (15 males and 5 females) were aged 69 ± 5 years (mean ± standard deviation). The mean degree of stenosis in the 40 carotids that were analysed was 53.1 ± 25.0%. In particular, the pre-procedure side showed 73.2 ± 5.2% stenosis, while the non-procedure side showed 33.0 ± 20.0% stenosis.

Table 1 reports the mean flow, peak velocity, and average cross-sectional area values in all patients for both 2D-PC and 4D-flow separated by branch. Pearson correlation coefficients *r*, as well as the *p*-values of the statistical analysis, were reported. There was a good correlation between the two techniques for the CCA flow (*r* = 0.65, *p* < 0.001), whereas for the ICA flow and ECA flow the correlation was only moderate (*r* = 0.4, *p* = 0.011 and *r* = 0.45, *p* = 0.003, respectively). The ECA flow was highly underestimated in the 4D-flow acquisition in comparison to the 2D-PC (2.5 mL/s vs. 1.9 mL/s, *p* = 0.020).

The correlations of peak velocities between methods were good for CCA (*r* = 0.56, *p* = 0.002) and moderate for ECA (*r* = 0.41, *p* = 0.008). There was no correlation for ICA (*r* = 0.04, *p* = 0.805). Peak velocities were significantly lower in the 4D-flow acquisitions in CCA (47.7 cm/s vs. 37.7 cm/s, *p* < 0.001), in ICA (41.1 cm/s vs. 25.9 cm/s, *p* < 0.001), and ECA (46.4 cm/s vs. 25.5 cm/s, *p* < 0.001).

Cross-sectional area values between methods showed no significant correlations for CCA (*r* = 0.18, *p* = 0.269), ICA (*r* = 0.1, *p* = 0.543), and ECA (*r* = 0.05, *p* = 0.767). The 4D-flow overestimated the vessel area in CCA (36.9 mm^2^ vs. 52.5 mm^2^, *p* < 0.001), in ICA (23.4 mm^2^ vs. 39.4 mm^2^, *p* < 0.001), and in ECA (20.0 mm^2^ vs. 35.9 mm^2^, *p* < 0.001).

**Figure 3:**
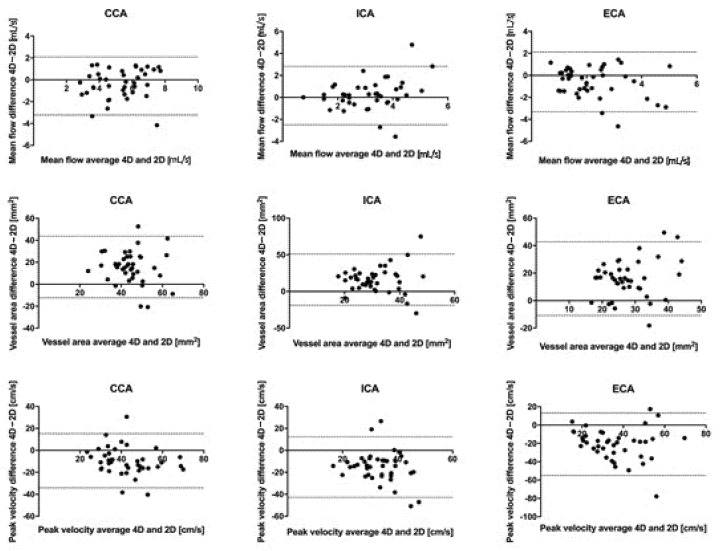
Bland-Altman plots of 4D-flow in all the carotid branches compared against 2D phase contrast. Dashed lines indicate the limits of agreement (±1.96 SD).

Bland-Altman plots of all variables are shown in Figure 3.

Data for the pre-procedure and non-procedure sides are presented separately in Table 2.

## 4. Discussion

Since the introduction of 4D-flow imaging, researchers have improved their ability to visualize complex vessel flows without the need for additional processes. This in vivo technique, which derived from the well-established 2D-PC, was applied in studies of ventricular flow [14], aortic flow [15], and even in relatively small vessels, such as the carotid arteries [16]. Furthermore, previous studies compared 4D-flow to DUS in the carotid arteries and to 2D-PC in the intracranial vasculature [17].

Flow comparisons between 4D-flow and 2D-PC showed a good to moderate correlation for the CCA and ICA, with 4D-flow systematically underestimating the flow rates in comparison to 2D-PC. The differences were only statistically significant for the ECA, probably due to the smaller dimension of this vessel. In fact, smaller vessels may be more difficult to segment due to a lower spatial resolution of 4D-flow. In addition, the lower spatial-temporal resolution of 4D-flow compared to 2D-PC and different semiautomatic algorithms of vessel segmentation can explain a systematic 4D-flow underestimation of the flow, albeit preserving a good correlation between the two methodologies. Since the presented results of mean flow (*Q*) were computed as the average of the product between the instantaneous velocity (*v*) and the cross-sectional area (*A*) (*Q* = *v* ∗ *A*), we should analyse the behaviour of these two variables separately.

Peak velocity was largely underestimated by the 4D acquisition, even though VENC was carefully chosen, avoiding aliasing over the whole course of the vessels for 2D acquisition. In the 4D acquisition, VENC was fixed, and we applied an antialiasing filter for post processing. As explained by Meckel et al. [17], lower peak velocities for 4D-flow are attributed to the significantly lower spatial-temporal resolution. In our case, temporal resolution between two consecutive acquisitions was the same between the two imaging methods, therefore the spatial resolution was the main cause of the difference. Indeed, pixel area was roughly four times higher in the 4D-flow as compared to the 2D acquisitions, creating an averaging effect similar to a low-pass filter, reducing the measured velocity. On the other hand, the slice thickness was higher in the 2D-PC compared to the 4D-flow. Of note, there was a good correlation for peak velocity in the CCA and ECA, whereas the ICA showed almost no correlation between 4D-flow and 2D-PC. We attributed this finding to the fact that the vessels analysed in this study displayed a wide range of stenosis (from 0% to 80%) and, in some cases, the lumen cross-sectional area had the same size as a single 4D-flow pixel, possibly leading to lower data quality in the presence of higher stenosis variability, such as in the ICA.

Cross-sectional areas were largely overestimated by 4D-flow imaging and correlation with 2D data was, in all cases, poor. Our measurements agreed with previously reported results of automated measurements in the CCA [18]. The reason for this could be explained by the influence of the noise or motion artefact in the automatic vessel segmentation. This result suggests caution in the interpretation of cross-sectional areas based solely on 4D-flow acquisitions and highlights the necessity of including sequences that allow a precise anatomical assessment in the acquisition protocol, such as black blood cine MRI sequences, which are known to minimise partial volume effects [19,20].

Finally, the data analysis spitted in pre-procedure and non-procedure sides showed no correlation between 2D-PC and 4D-flow for pre-procedure ICA and of peak velocity for pre-procedural CCA. In all the other comparisons, no difference in correlation between side was found. This result suggests that severe carotid stenosis does not worsen 4D-flow assessment.

Our work has some limitations. First of all, the carotid MR within our study was performed as a part of a clinical trial in patients that were screened for carotid artery stenosis. Therefore, we proposed an MR protocol with a reasonable compromise between scan duration and image quality. Most of the published studies included a dedicated, experimental 4D sequence lasting 20–30 min, which would sometime also involve contrast-agent intravenous injection. In our protocol, the duration of the 4D-flow sequence itself was below 15 min (with a reduction of spatial resolution in favour of temporal resolution and a large field of view), to render such acquisitions more suitable for clinical practice. We also opted for a coronal acquisition to ensure the visibility of the entire carotid extension, from its origin in the arch to the intracranial vasculature. Therefore, while other studies report higher spatial and temporal resolution for 4D-flow [12], scan duration may be more than double, thus being unsuitable as a routine clinical approach.

Secondly, the choice of the analysis plane in the 4D images was performed manually by an experienced operator to be as close as possible to the level of the 2D acquisition. However, this could have introduced some errors, namely in the velocity and area measurements. A dedicated image co-registration software would be ideal to overcome this issue.

## 5. Conclusions

In conclusion, 4D-flow imaging provided a good and moderate correlation of flow rates with 2D-PC for the CCA and ICA, respectively. Conversely, peak velocity and average area measurements were, respectively, underestimated and overestimated by 4D-flow imaging, and therefore need to be carefully interpreted in the context of the lower spatial and time resolution of 4D-flow. With a fast 4D-flow protocol, it might be possible to gather more information regarding CCA and ICA flow before and after treatment. These flow data could be helpful in the managing of carotid treatment.

## Figures and Tables

**Table 1:** Patients’ mean flow, peak velocity, and average cross-sectional areas.

	Mean Flow [mL/s]				Peak Velocity [cm/s]				Average Cross-Sectional Area [mm^2^]			
Vessel	2D-PC	4D-Flow	Pearson *r*	*p*-Value	2D-PC	4D-Flow	Pearson *r*	*p*-Value	2D-PC	4D-Flow	Pearson *r*	*p*-Value
CCA	5.5 ± 1.5	5.1 ± 1.7	0.65,	0.124	47.7 ± 14.7	37.7 ± 12.0	0.56,	<0.001	36.9 ± 12.0	52.5 ± 10.8	0.18,	<0.001
			*p* < 0.0001				*p* = 0.0002				*p* = 0.269	
ICA	3.0 ± 1.1	3.3 ± 1.4	0.40,	0.221	41.1 ± 11.3	25.9 ± 8.8	0.04,	<0.001	23.4 ± 11.5	39.4 ± 12.5	−0.1,	<0.001
			*p* = 0.011				*p* = 0.805				*p* = 0.543	
ECA	2.5 ± 1.5	1.9 ± 1.2	0.45,	0.020	46.4 ± 17.2	25.5 ± 14.7	0.41,	<0.001	20.0 ± 8.6	35.9 ± 11.0	0.05,	<0.001
			*p* = 0.003				*p* = 0.008				*p* = 0.767	

Values are reported as mean ± standard deviation. The *p*-value corresponds to the comparison between groups. 2D-PC: 2D phase contrast; CCA: common carotid artery; ICA: internal carotid artery; ECA: external carotid artery.

**Table 2:** Mean flow, peak velocity, and average cross-sectional area for the pre-procedure and non-procedure side in the study population.

		Flow [mL/s]				Peak Velocity [cm/s]				Cross-Sectional Area [mm^2^]			
		2D-PC	4D-Flow	Pearson *r*	Wilcoxon *p*-Value	2D-PC	4D-Flow	Pearson *r*	Wilcoxon *p*-Value	2D-PC	4D-Flow	Pearson *r*	Wilcoxon *p*-Value
CCA	Pre-procedure	5.1 ± 1.3	4.7 ± 1.6	*r* = 0.746, *p* < 0.001	*p* = 0.332	47.5 ± 12.8	36.8 ± 11.0	*r* = 0.368, *p* = 0.111	*p* = 0.002	33.4 ± 8.8	51.3 ± 11.7	*r* = 0.259, *p* = 0.270	*p* < 0.001
	Non-procedure	5.8 ± 1.6	5.6 ± 1.6	*r* = 0.647, *p* = 0.002	*p* = 0.601	48.0 ± 16.7	38.6 ± 13.2	*r* = 0.602, *p* = 0.005	*p* = 0.004	40.4 ± 13.9	53.6 ± 10.0	*r* = 0.081, *p* = 0.734	*p* = 0.006
ICA	Pre-procedure	2.6 ± 1.2	2.8 ± 1.4	*r* = 0.335, *p* = 0.148	*p* = 0.502	42.7 ± 12.0	23.6 ± 8.2	*r* = 0.368, *p* = 0.111	*p* < 0.001	21.4 ± 9.5	39.6 ± 14.7	*r* = 0.077, *p* = 0.748	*p* < 0.001
	Non-procedure	3.4 ± 0.8	3.7 ± 1.3	*r* = 0.466, *p* = 0.038	*p* = 0.167	39.5 ± 10.7	28.2 ± 9.1	*r* = 0.090, *p* = 0.705	*p* = 0.005	25.4 ± 13.2	39.3 ± 10.2	*r* = 0.236, *p* = 0.316	*p* = 0.005
ECA	Pre-procedure	2.7 ± 1.7	1.7 ± 1.1	*r* = 0.268, *p* = 0.254	*p* = 0.011	46.3 ± 19.6	24.1 ± 15.8	*r* = 0.574, *p* = 0.008	*p* < 0.001	21.7 ± 10.8	36.3 ± 9.4	*r* = 0.134, *p* = 0.574	*p* = 0.001
	Non-procedure	2.4 ± 1.2	2.2 ± 1.2	*r* = 0.349, *p* = 0.132	*p* = 0.550	46.5 ± 14.9	27.0 ± 13.7	*r* = 0.569, *p* = 0.009	*p* < 0.001	18.2 ± 5.5	35.6 ± 12.7	*r* = 0.131, *p* = 0.582	*p* < 0.001

Values are reported as mean ± standard deviation. The *p*-value corresponds to the comparison between groups. 2D-PC: 2D phase contrast; CCA: common carotid artery; ICA: internal carotid artery; ECA: external carotid artery.

## Data Availability

The data presented in this study are available on reasonable request from the corresponding author. The data are not publicly available due to privacy concerns.
